# Alginate-encapsulated muscle-derived stem cell spheroids promote muscle regeneration in a murine model of volumetric muscle loss

**DOI:** 10.3389/fphar.2025.1657563

**Published:** 2025-12-12

**Authors:** Lucas Pari Mitre, Giovana Zanetti Ivanov, Shahin Shams, Fabio Yamaguchi, André Petraconi, Ricardo Jorge Espanhol Andrade, Eduardo Silva, Leonardo Martin, Roberta Sessa Stilhano

**Affiliations:** 1 Department of Physiological Sciences, Santa Casa de São Paulo School of Medical Sciences, São Paulo, Brazil; 2 Post-graduation Program of Chemistry Biology, Federal University of Sao Paulo, Diadema, Brazil; 3 Biomedical Engineering Department, University of California, Davis, CA, United States; 4 Traumatology and Orthopedics Institute, University of São Paulo, São Paulo, Brazil; 5 MackGraphe-Mackenzie Institute for Research in Graphene and Nanotechnologies, Mackenzie Presbyterian Institute, Sao Paulo, Brazil; 6 Engineering School, Mackenzie Presbyterian University, São Paulo, Brazil; 7 Department of Chemistry, Bioscience, and Environmental Engineering, University of Stavanger, Stavanger, Norway; 8 Department of Pharmaceutical Sciences, University of Antwerp, Antwerp, Belgium; 9 Center of Excellence Infla-Med, University of Antwerp, Antwerp, Belgium

**Keywords:** muscle injury, muscle derived stem cells, alginate, volumetric muscle loss, spheroids

## Abstract

**Introduction:**

Volumetric muscle loss (VML) remains a major clinical challenge due to the limited capacity of skeletal muscle to regenerate large-scale injuries. Muscle-derived stem cells (MDSCs) represent a promising therapeutic option for tissue regeneration; however, their clinical application is constrained by poor post-transplantation viability and limited engraftment. Alginate hydrogels offer a supportive three-dimensional microenvironment capable of encapsulating cells, promoting their survival, and enhancing paracrine signaling through the sustained release of growth factors.

**Methods:**

In this study, we developed and characterized MDSC spheroids and evaluated their regenerative potential when encapsulated in RGD-modified alginate hydrogels. Co-culture with endothelial cells significantly enhanced spheroid viability, indicating beneficial paracrine interactions. To further refine this strategy, 5% of the MDSCs were preconditioned with vascular endothelial growth factor (VEGF) prior to spheroid formation and encapsulation, integrating a pharmacological preconditioning step into the cell–hydrogel platform. Encapsulated spheroids were implanted into a murine model of VML.

**Results:**

After 30 days, animals treated with alginate-encapsulated MDSC spheroids containing a 5% VEGF-preconditioned subfraction exhibited reduced granulation tissue, fewer degenerating myofibers, lower fibrosis, and improved early rota-rod performance compared with untreated and scaffold-only controls.

**Discussion:**

Together, these findings highlight a pioneering proof-of-concept platform that combines 3D MDSC spheroids, alginate-based delivery, and VEGF-mediated pharmacological preconditioning for VML repair. As a 100% unconditioned MDSC+alginate group was not included, the present study should not be interpreted as demonstrating *in vivo* superiority of VEGF preconditioning over unconditioned MDSCs; instead, it provides a rationale for future head-to-head studies explicitly powered to address this question.

## Graphical Abstract

**Figure:**
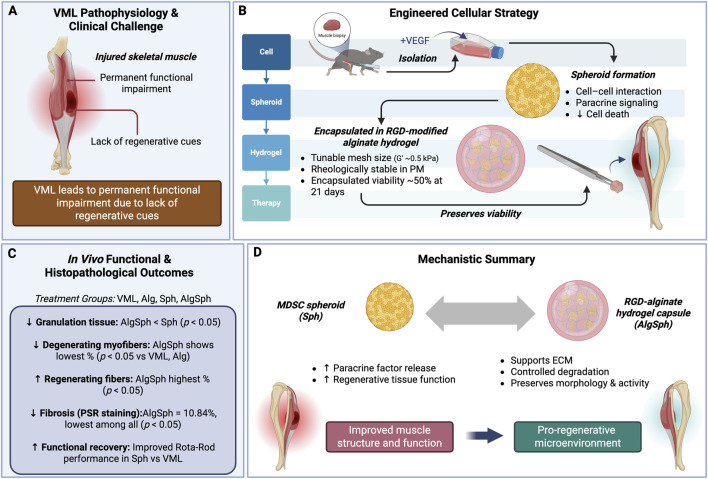

## 1 Introduction

Volumetric Muscle Loss (VML) remains an underwhelming public health concern. Lack of relevant epidemiological data undermines measurement of real-word impact of VML on disability adjusted life years (DALY) and quality adjusted life years (QALY). Muscle loss primarily affects muscle tissue by leading to fibrosis and atrophy. Despite the musculoskeletal regeneration properties, certain types of muscle injury prove to lead to stochastic damages and ultimately irrecoverable loss of function (Greising et al., 2016).

Pathological mechanisms behind VML injury encompass alterations in myocyte microenvironment, excessive extracellular fibrillar deposition in response to injury and loss of cellular components (Corona et al., 2016). One key mechanism behind VML injury perpetuation includes loss of satellite cells. A special niche of satellite cells, named muscle derived stem-cell like cells (MDSC) play an important role in tissue engineering strategies (Gharaibeh et al., 2008; Vojnits et al., 2015).

Beyond stem cell depletion, VML’s alteration in macrophage lineages dynamics predispose muscle to fibrosis post injury. Imbalances between M1 and M2 macrophages may lead to excessive deposition of extracellular matrix and proinflammatory cytokines, favoring sarcopenia, fibrosis, atrophy and an overall myocytotoxic environment (Prisk and Huard, 2003; Peng and Huard, 2004).

In clinical practice, VML injuries usually are present in trauma and war scenarios. Thus, patients with VML injuries are first seen by emergency medicine doctors and orthopedic surgeons (Garg et al., 2015; Corona et al., 2016). In acute care scenarios, VML injuries are most likely managed with suturing of superficial tissues, avoiding exposure of the noble triad of muscle: vessels, tendons and nerves (Garg et al., 2015; Corona et al., 2016). Less likely, VML injuries are offered with the counterpart of muscle flap transfer, when not delayed by complications. Flaps are a viable solution, but inherently limit the therapeutic options by offering risks to both donor and receptor sites, not completely promoting muscle regeneration (Garg et al., 2015; Quarta et al., 2017).

Bioengineering strategies are an interesting option considering the scarcity of muscle availability and technical training for executing flaps excision and suturing, especially in acute care scenarios. Those strategies involve utilizing biomaterials enhanced with cellular or matrix components. In the musculoskeletal system, myocyte-enriched matrices and biomaterials are able to reproduce contractile function (Corona et al., 2013; Li et al., 2023; Rousseau et al., 2023). Biomaterials represent an interesting strategy for modulating inflammation, stem cell differentiation fates and both cellular-cellular and cellular-matrix interactions, favoring survival and remodeling (Wu et al., 2025). Material-specific competence in carrying factors and exposing extracellular signals mimicking native cellular environment. Alginate’s capability of encapsulating cells proves interesting in constituting cellular delivery strategies in vascular, neoplastic and genetic diseases (Silva and Mooney, 2007; 2010; Campbell et al., 2018). Tunability of alginate hydrogel in terms of integration of common cellular adhesion proteins into extracellular matrix allows for 3-dimensional cellular-cellular and cellular-extracellular matrix interaction. Common examples utilized in this setting include integrins, such as the RGD peptide (a fraction of mammalian integrin), and YAP protein. Besides quality of ligands, tunability can occur with control of the degree of substitution, considering alginate hydrogel’s polymer structure. It is also known that degree of substitution of same ligands influences the strength of cellular adhesion and, thus, intracellular processes response (Gionet-Gonzales et al., 2023).

Exploring cellular-cellular interaction, spheroids represent a disruptive cellular culture technique. Scaffold free methods allow for strong intracellular adhesion development, mimicking the *in vivo* dimensionality with better precision (Stuart et al., 2017). Ultimately, the effect of bidimensional nuclear differential genetic expression is attenuated (Rashedi et al., 2017).

Vascular support is a critical determinant of VML repair, and VEGF (vascular endothelial growth factor) occupies a central position at this interface. Skeletal muscle homeostasis and regeneration depend on intimate crosstalk between myogenic and endothelial compartments; capillary rarefaction and impaired angiogenic signaling hinder graft survival and integration after large-volume loss (Latroche et al., 2017). VEGF orchestrates endothelial survival, proliferation, and tube formation and, crucially for cell therapy, can promote cytoprotection of myogenic progenitors, improving resistance to stress during delivery and early engraftment (Nakayama et al., 2019; Verma et al., 2024). Prior studies demonstrating improved outcomes with endothelial-directed programming of myogenic cells in ischemic contexts.

Many studies evaluated the response to muscle injury to cellular therapy (Andrade et al., 2015; Helal et al., 2016; Rashedi et al., 2017; Ichiseki et al., 2023). To this day, on the other hand, few have examined the interplay between 3-dimensional cellular culture encapsulation in alginate hydrogels for therapeutics in VML injury (Gionet-Gonzales et al., 2023; Wu et al., 2025). Our goal was to develop a delivery strategy of cellular therapy to VML lesions by encapsulating a mixture of naïve MDSCs and VEGF-pre-treated MDSCs spheroids within rheologically optimized alginate hydrogels. We then evaluated the therapeutic efficacy of this platform *in vivo* using a murine model of VML.

## 2 Materials and methods

### 2.1 Cell culture

MDSC primary culture was established as previously described protocols (Gharaibeh et al., 2008). Primary MDSCs were isolated from pooled skeletal muscles of 1-month-old C57BL/6 mice. The MDSC population was expanded up to passages 3–5 and cryopreserved in aliquots to establish a working cell bank, which was subsequently used for all *in vitro* and *in vivo* experiments. All animal procedures were approved by the institutional ethics committee (Protocol 003/22; June 6, 2022). Human endothelial HUVEC cells were kindly provided by Prof. Dr. Sang Won Han (UNIFESP, São Paulo, Brazil). Two HUVEC preparations were used in this study: i. a GFP-transduced HUVEC line generated by lentiviral transduction (Lv-VEGF-GFP) that was used exclusively in preliminary mixing controls to confirm the targeted 95:5 MDSC:HUVEC composition by fluorescence microscopy; and ii. the parental, non-transduced HUVEC line, which was used for all experiments reported in the manuscript, including spheroid formation, Live/Dead viability assays (Calcein-AM/7-AAD), and any downstream analyses. No GFP-expressing HUVECs were included in viability quantification, thereby avoiding any potential interference with the green fluorescence channel. Unless otherwise specified, MDSC were cultivated in Proliferation Medium (PM), constituted by Dulbecco’s modified Eagle’s medium (DMEM; Thermo Fisher Scientific, Inc., United States) supplemented with 10% Fetal Bovine Serum (FBS, Thermo Fisher), 10% Horse Serum (HS, Thermo Fisher), 0,5% Chicken Embryo Extract (MP Biomedicals, United States) and 1% Penicillin/Streptomycin (Thermo Fisher). Endothelial differentiation followed the protocol previously described, with modifications (Harding et al., 2017). Cells were seeded on plates covered with type I bovine collagen (Thermo Fisher) and cultured with endothelial cell growth media (EGM, Endothelial Cell Growth Medium-2, Lonza) supplemented with 50 ng/mL of VEGF (Peprotech) for 7 days. All cellular cultures were incubated at 37 °C with an atmosphere of 5% CO_2_ (MCO-170AICUV(H)L-PA incubator, Panasonic, Japan) and tested free of *mycoplasma* contamination. All cellular culture manipulations were conducted in a sterile laminar-flow environment.

### 2.2 3D Cell culture model

The spheroids were synthesized as previously described (Seixas et al., 2023). In summary, 1 × 10^6^ trypsinized cells of designed cellular cultures were added into each micromold, yielding 81 spheroids, with an average of 1.2 × 10^4^ cells/spheroid. Each mold was maintained with 2 mL of appropriate culture media. For MDSC/HUVEC spheroids, a mixture of 95% MDSC and 5% HUVECs was achieved before pipetting the cells into molds. *In vivo* therapeutic spheroids constituted a mixture of 95% MDSC and 5% MDSC-VEGF (MDSC/MDSC-VEGF), which were pre-differentiated into endothelial lineages through cell culture in EGM-VEGF enriched media for 7 days (50 ng/mL VEGF). All *in vitro* spheroid assays were followed for 21 days. For *in vivo* therapeutic use, MDSC/MDSC-VEGF spheroids were synthesized by mixing 95% MDSCs and 5% VEGF-preconditioned MDSCs, and used within 24 h of formation. These spheroids were encapsulated on day 1 of culture to preserve viability and functional phenotype. Spheroid DNA quantification was assessed through Quant-iT™ PicoGreen™ (Thermo Fisher, United States) for such *in vivo* spheroids. For each timepoint, independent vials containing multiple spheroids were processed in parallel, and total DNA was extracted from each vial as a single sample. The resulting DNA quantity was then normalized by the number of spheroids within that vial to derive the value per spheroid. This approach results in multiple measurements per timepoint that originate from biologically independent vials, rather than technical replicates of the same sample. All spheroids were periodically assessed for size (longitudinal diameters) and morphology under bright-field microscopy with Axio Vert.A1 microscope (ZEISS, Germany). Sphericity was defined as the ratio of the vertical over horizontal longitudinal ratios for a specific spheroid. Microphotographic acquisition was followed with vertical and horizontal radius measurement (“Straight, segmented or freehand lines” tool) utilizing ImageJ (National Institute of Mental Health, United States). The same cellular population and quantity designated to formulate the spheroids was plated, as described in *Cell culture, and* 2-dimensional microphotographic acquisition of adherent cells was performed, to illustrate, in Figure 2, the difference in the size of spheroids not being related to a significant difference in individual cellular size.

### 2.3 Alginate hydrogel encapsulation assay

Low molecular weight alginate (MW 50 kDa) used in this study was obtained from Novamatrix (Norway). Alginate peptide residue modification with RGD (GGGGRGDSP-COOH, AminoTech, Brazil) was achieved through carbodiimide chemistry, yielding a degree of substitution of 2. The spheroids were encapsulated in RGD-modified low molecular weight, 0% oxidized, alginate hydrogel, as previously described, with a ratio of 2% weight/volume (w/v) (Whitehead et al., 2021). Spheroids were mixed with unpolymerized alginate. For CaCl_2_ gelification assays, the spheroid-alginate mixture was pipetted with a 1,000 µm pipette tip onto a 200 mM CaCl_2_ bath, forming macroscopic alginate hydrogel spheres, with a goal of 3 spheroids per hydrogel. The hydrogel spheres were then strained and washed 3 times with sterile normal saline (NaCl 0.9%). A batch of cell-free alginate hydrogels was synthesized, utilizing the same gelation method, for quantifying the cellular impact on hydrogels rheological properties. All hydrogels were cultivated under different culture media at 37 °C and 5% CO_2_. Ten spheroids were manually transferred into each hydrogel under sterile conditions using micropipette handling for *in vivo* experimenting. The number of spheroids per hydrogel was confirmed by direct visual inspection under bright-field microscopy prior to implantation.

### 2.4 Rheological assays

Every gel was assessed for its wet weight right after culture media suspension, and posteriorly, for its dry weight, after 24 h of lyophilization. Swelling ratio (Q) was calculated through the formula Q = ((W_s_-W_d_/P_w_) + (W_d_/P_p_))/(W_d_/P_p_) where W_s_ is wet weight, W_d_ is dry weight, P_w_ is density of the water and P_p_ is density of the polymer. Mesh size was measured through interpretation of swelling data. Average mesh size of the network structure was calculated via Canal-Peppas equation, the derived formula of Flory–Rehner theory for anionic polymers: Mesh size = ξ = Q^1/3^ * l * (2M_c_/M_r_)^1/3^ * C_n_^1/2^, where M_c_ is molecular weight between crosslinks, l is the length of the repeating unit and M_r_ is the molecular weight of the repeating unit, and C_n_ is the characteristic ratio of the polymer. M_c_ was obtained from the formula derived for anionic alginate polymers: M_c_ = C_p_*R*T/G′, where C_p_ is the polymer concentration, R is the gas constant, T is temperature and G′ is the storage modulus. For G′ and G″ measurement, alginate hydrogels were submitted to a range of strain of (0.01%–10%) at a constant (10 rad/s), and the tests were performed 1 day after alginate polymerization, in a Anton Paar MCR 702 rheometer, and an average of 12-point analysis was obtained. Temperature was controlled at 25 °C, and rheological analysis was conducted under 8 mm parallel plates. At each timepoint, excess hydrogels were generated, and representative samples were selected based on structural integrity to maximize sample size (n) while maintaining experimental consistency. Further rheological parameters were previously described (Campbell et al., 2018).

### 2.5 Immunofluorescence and viability assays

Fluorescence microscopy images were acquired to assess cellular viability. Live/Dead assay was performed with 7-AAD (7-Aminoactinomycin D) and Calcein-AM (Thermo Fisher). Fluorescence imaging was obtained utilizing Leica DMi8 microscope (Leica Microsystems, Germany). Spheroids’ cellular viability was assessed through the corrected total cell fluorescence (CTCF) method. After image acquisition, green and red split channels of total cellular fluorescence were quantified with ImageJ (National Institute of Mental Health, Bethesda, Maryland, United States) for integrated density measurement. Three random background areas for each spheroid image were acquired for subtracting background fluorescence, according to the formula: CTCF = Integrated Density – (Area of selected spheroid x Mean fluorescence of background readings). Each spheroid viability (%) was determined following the formula: CTCF (viability, %) = CTCF green/(CTCFgreen+CTCF red).

### 2.6 Gene expression assessment via real-time quantitative polymerase chain reaction (RT-qPCR)

Total RNA from cultured cells was extracted with Trizol reagent (Life Technologies). The cDNA was obtained from the extracted RNA using the High-Capacity Kit (High-Capacity cDNA Reverse Transcription Kit, Applied Biosystems^TM^ - Thermo Fisher) according to the manufacturer’s instructions. The primers and probes utilized for the RT-qPCR were: Ly6a_mouse_F CCT ACC CTG ATG GAG TCT GTG T, Ly6a_mouse_R CAC GTT GAC CTT AGT ACC CAG G, R18S_F GCC GCT AGA GGT GAA ATT CT, R18S_R CGA ACC TCC GAC TTT CGT TC and Quanti-Nova SYBR-Green (Qiagen). The reaction was performed in Quanti-Studio (Applied Biosystems), and was analyzed by a relative comparison method (2^−ΔΔCt^). RNA extraction from 3D alginate-encapsulated spheroids was piloted but yielded insufficient, inhibitor-free RNA at the required implantation timepoints. Despite alginate lyase digestion and additional purification, polymer carryover impaired downstream RT-qPCR. To avoid delaying time-sensitive implantation, stemness assays were therefore performed on early-passage 2D MDSCs; spheroid-level profiling is planned in future work.

### 2.7 Volumetric loss injury model

All animal procedures were approved by the institutional ethics committee (Protocol 003/22; June 6, 2022) and reported in accordance with ARRIVE 2.0. Three-month-old male C57BL/6 mice (n = 30; 25–28 g) were obtained from CEDEME (Center for the Development of Experimental Models in Medicine and Biology, Brazil). Volumetric muscle loss (VML) was induced in the left gastrocnemius under anesthesia with ketamine (100 mg/kg) and xylazine (10 mg/kg), following a previously described procedure with minor modifications (Hu et al., 2025; Greising et al., 2018; Basten et al., 2023). Briefly, the muscle was surgically exposed and a 4-mm circular defect was created using a sterile biopsy punch (Rhosse Instrumentos e Equipamentos Cirúrgicos EIRELI EPP, Brazil). Animals were identified by ear notching. For treatment, alginate hydrogel–encapsulated spheroids, cell-free alginate hydrogels, or spheroids alone were applied to the defect; a sham surgery group served as control. The muscle fascia, subcutaneous tissue, and skin were then closed with PDS II sutures (Ethicon, Inc.).

### 2.8 Rota-rod functional assay

Muscle performance was assessed 15 and 30 days postoperatively, utilizing RotaRod testing (RotaRod, EFF 411, Insight, Brazil). The animals were familiarized with a period of training on the device in the 2 days prior to the test days, being submitted to 2 races of 3 min at 24 RPM, being removed within each fall. On the day of the test, the animals were placed in the RotaRod with an initial speed of 10 RPM, progressing to 24 RPM over 5 min (Felipone et al., 2025). The fall time was recorded for each animal individually and displayed graphically as individual plotted data for each group (encapsulated spheroids, cell-free hydrogels, spheroids alone and sham surgery).

### 2.9 Histology

Thirty days following the surgical procedure and after RotaRod testing, animals were euthanized for the evaluation of muscle regeneration by histological analysis. Gastrocnemius muscles were harvested, fixed in 4% paraformaldehyde, embedded in paraffin, and sectioned for staining with Hematoxylin and Eosin (H&E) and Picrosirius Red. Image acquisition and quantification were performed using ImageJ and ImagePro Plus (Media Cybernetics, Rockville, MD, United States). To account for inter-sample variability, all histomorphometric measurements were normalized to the injury area of each individual sample, and data were expressed as the percentage of each histological parameter relative to the total injury area. The histological evaluation encompassed the analysis of granulation tissue development, the extent of muscle fiber degeneration and regeneration, and collagen deposition within the injured tissue. Fibrotic areas were defined by collagen accumulation in the endomysial and perimysial spaces between muscle fibers, as visualized by Picrosirius Red staining. The region of interest (ROI) was delineated within the central and regenerative zones of the injury site, deliberately excluding the survival zone and the epimysial cap (Figure 5C), in accordance with anatomical definitions established by Hurme et al. (1991). Quantification of fibrotic tissue was adapted from the methodology described by Martins et al. (2014), while the overall histomorphometric criteria were based on the protocol recently validated by Felipone et al. (2025). For samples with near-complete remodeling at Day 30, the ROI was confined to the central/regeneration zones (Figure 7C), excluding survival zone/epimysial cap; injured area extent was also measured and is reported in Supplementary Table S1 to document differences in residual lesion size. Additional details regarding the histopathological evaluation criteria can be found in the Supplementary Material (Supplementary Figure S2; Supplementary Table S1).

### 2.10 Statistical analysis

Unless specified, the results obtained are herein expressed as mean ± SD (standard deviation) and submitted to statistical analysis. Statistical analysis was performed by one-way ANOVA with Sidak’s multiple comparisons test or Mann-Whitney test when appropriate. The software used was the GraphPad Prism version 9.0 (GraphPad Software, Inc., La Jolla, CA, United States). The results obtained were considered statistically significant if p < 0.05. Different letters imply statistical significance.

## 3 Results

### 3.1 MDSC exhibit increased stemness potential when cultivated in endothelial culture media

MDSC were cultivated in PM on low confluency (<30%) for stem cell properties maintenance (Supplementary Figure S1A). When MDSC reached higher confluences (60%), clustered differentiation was observed under bright-field microscopy. The visual aspect of round, small cells with greater nuclei proportion was typical of MDSC with preserved stemness. Fusiform, bundled group of cells with multinucleated fibers was typical of terminal differentiation (Supplementary Figure S1B). To quantify stem cell potential, RT-qPCR was performed for key transcription factors. Sca-1 (*stem cell antigen-1 - Ly6a)* was assessed as a proxy for stemness. Ly6a expression was 3-fold higher in MDSC cultured in EGM media in comparison to PM (Supplementary Figure S1C, p < 0.05).

### 3.2 Coculture of MDSC with endothelial cell lineages produced 3-D morphological and growth changes in spheroids

**FIGURE 1:**
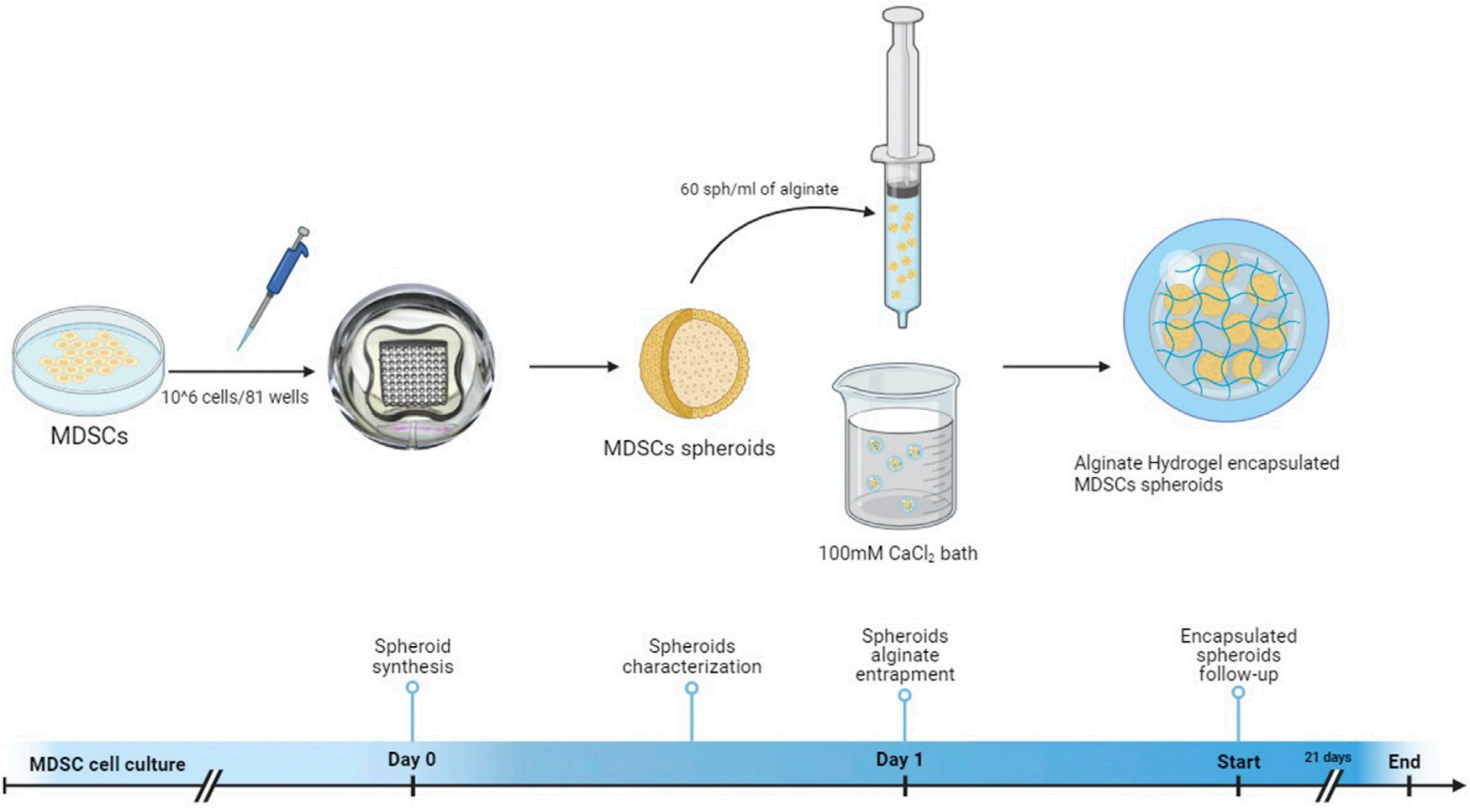
Schematization of the workflow illustrating spheroid synthesis and encapsulation into CaCl_2_-polymerized hydrogels. Schematic illustration of study design. Created with BioRender.com.

**FIGURE 2:**
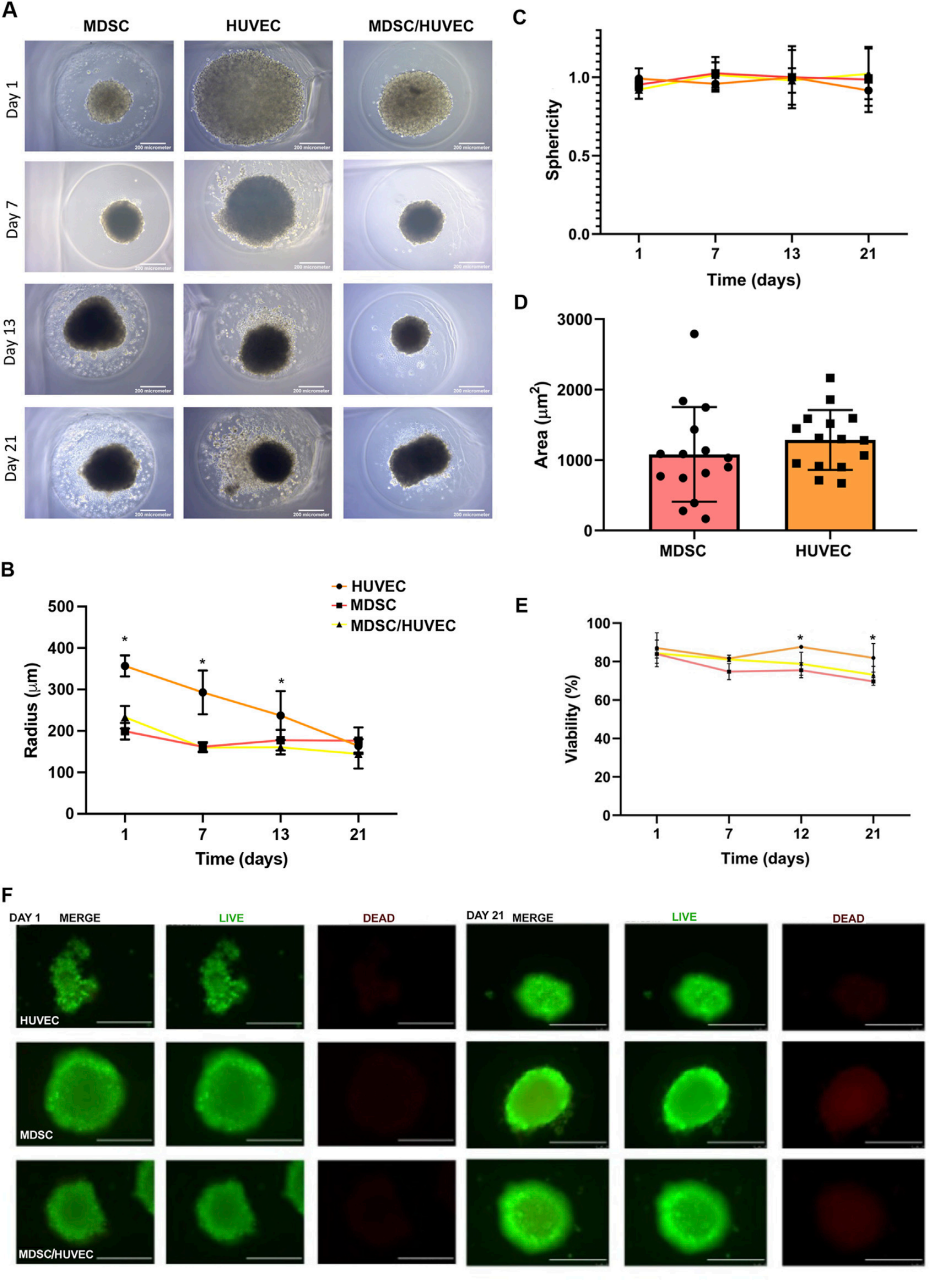
Spheroid synthesis and characterization. **(A)** Representative bright-field images of spheroids over 21 days of experiment. **(B)** Comparison of the radius of MDSC, HUVEC, and MDSC/HUVEC spheroids over time. Day 1: *p < 0.05 (HUVEC X MDSC), (HUVEC X MDSC/HUVEC) and (MDSC X MDSC/HUVEC), Day 7: *p < 0.05 (HUVEC X MDSC), (HUVEC X MDSC/HUVEC), Day 13: *p < 0.05 (HUVEC X MDSC/HUVEC). **(C)** Comparison of the sphericity of MDSC, HUVEC, and MDSC/HUVEC spheroids over time. **(D)** MDSC and HUVEC bi-dimensional area on traditional plating method. This area pertains to the same cellular quantity and individual cellular area displayed when plated and adhered bidimensionally, not to the bidimensional area of the spheroid. **(E)** Spheroids viability quantification assessed through Live/Dead assay, over 21 days of experiment. Day 12: *p < 0.05 (HUVEC X MDSC/HUVEC), Day 21: *p < 0.05 (HUVEC X MDSC/HUVEC). **(F)** Representative spheroids Live/Dead assay images. Scale bar = 200 µm.

We initially conducted a morphological and growth characterization of MDSC, HUVEC, or a mixture of MDSC/HUVEC (95:5) spheroids before their encapsulation with alginate. A workflow illustrating the synthesis, culture, and encapsulation process of MDSC spheroids is depicted in Figure 1. All three spheroid groups underwent the same synthesis process to evaluate baseline morphological characteristics over the 21-day experimental period while being cultured in endothelial growth media (EGM). All spheroids exhibited a spherical morphology and homogeneity (Figure 2A). Despite variations in radius over time (Figures 2A,B), all spheroids maintained their spherical shape throughout the 21 days (Figure 2C).

MDSC spheroids showed a bimodal growth pattern, with an initial mean radius of 199.3 ± 2.18 µm. They experienced an initial decrease of 18.86% in mean radius from day 1 to day 7, followed by a subsequent 9.09% increase until day 21. By the end of the experiment, the mean overall radius decreased by 11.49% (Figure 2B).

HUVEC spheroids exhibited distinct behavior, with an average radius double that of MDSC spheroids, measuring 356.9 ± 25.55 µm, which continuously decreased throughout the 21-day experiment period, with a mean total radius decrease of 54.02% (Figure 2B). No differences were detected between the areas occupied by MDSC and HUVEC under conventional cell culture conditions (Figure 2D).

MDSC/HUVEC spheroids initially had a radius averaging between MDSC and HUVEC spheroids, at 233.2 ± 27.25 µm, and were statistically different from both MDSC and HUVEC on day 1 (Figure 2B, p < 0.05). However, as the experiment progressed, MDSC/HUVEC spheroids tended to behave more similarly to MDSC spheroids, with no statistically significant difference observed at the end of the experiment (Figure 2B).

The Live/Dead assay of MDSC/HUVEC spheroids revealed a mean viability of 87.05% ± 7.915% for HUVEC spheroids, 83.938% ± 2.084% for MDSC spheroids, and 82.53% ± 6.978% for MDSC/HUVEC spheroids on day 1, with no initial statistical differences observed (Figures 2E,F). However, MDSC/HUVEC spheroids exhibited superior viability on day 7 compared to MDSC spheroids, with viabilities of 81.113% ± 0.850% and 74.813% ± 4.15%, respectively (Figures 2E,F). The superior viability of MDSC/HUVEC spheroids remained consistent until the end of the experiment, although not statistically significant.

### 3.3 Long-term stability of alginate hydrogel physical parameters in different cell culture media

**FIGURE 3:**
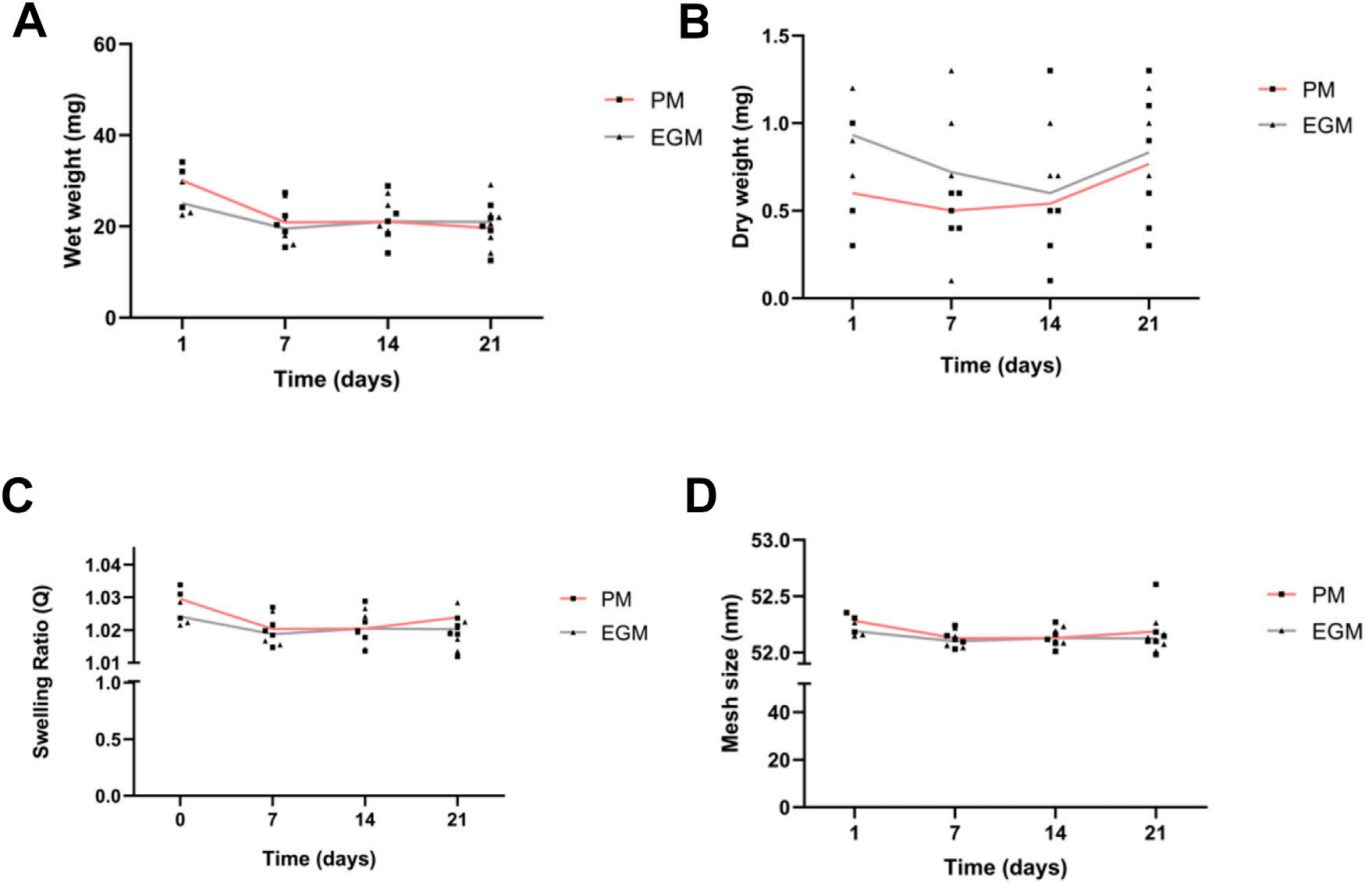
RGD-modified LMW alginate hydrogels characterization. CaCl_2_-polymerized alginate hydrogels and physical measurements over time. **(A)** Wet weight. **(B)** Dry weight. **(C)** Swelling Ratio. **(D)** Mesh size.

To evaluate the characteristics of the alginate hydrogel prior to spheroid encapsulation, we monitored its rheometric parameters while immersed in different cell culture media. The clean alginate hydrogels exhibited consistent wet and dry weights throughout the 21-day experiment period, regardless of the cell culture media used. Initially, the mean wet weights of the hydrogels were 30.10 ± 5.216 mg for PM and 25.10 ± 4.078 mg for EGM, with corresponding mean wet weights at day 21 of 19.60 ± 4.491 mg for PM and 20.983 ± 5.071 mg for EGM. Statistical analysis revealed no significant differences between the PM and EGM groups at any time point (Figure 3A). Wet and dry weight (Figures 3A,B) stability led to a stable swelling ratio, with an initial mean of 1.03 ± 0.005 for PM, and 1.024 ± 0.004 for EGM, and a day 21 mean of 1.024 ± 0.013 for PM and 1.020 ± 0.005 for EGM (Figure 3C). Ultimately, our alginate hydrogel sphere had an initial mean mesh size of 52 nm on average, more specifically, 52.281 ± 0.088 nm for PM and 52.191 ± 0.065 nm for EGM (Figure 3D), serving as an initial baseline characterization. Comparing the rheological data obtained from our hydrogel, the hydrogel’s Loss Modulus approximated closely to human gastrocnemius Loss Modulus *in vivo* rheological assay measurements (Table 1) (Green et al., 2012).

**TABLE 1:** Comparison of alginate hydrogels and human muscle rheometric variables.

Sample	Loss modulus (kPa)	Storage modulus (kPa)	Shear stress (kPa)
Alginate hydrogel*	0.500 ± 0.15	4.67 ± 0.89	0.013 ± 0.018
Green et al. (2012)	0.44 ± 0.12	1.15 ± 0.23	NR

*this data was quantified over 12 gels (n=12).

### 3.4 Encapsulated MDSC spheroids induced rheometric changes on alginate polymeric structure, and are able to maintain cellular viability over time

**FIGURE 4:**
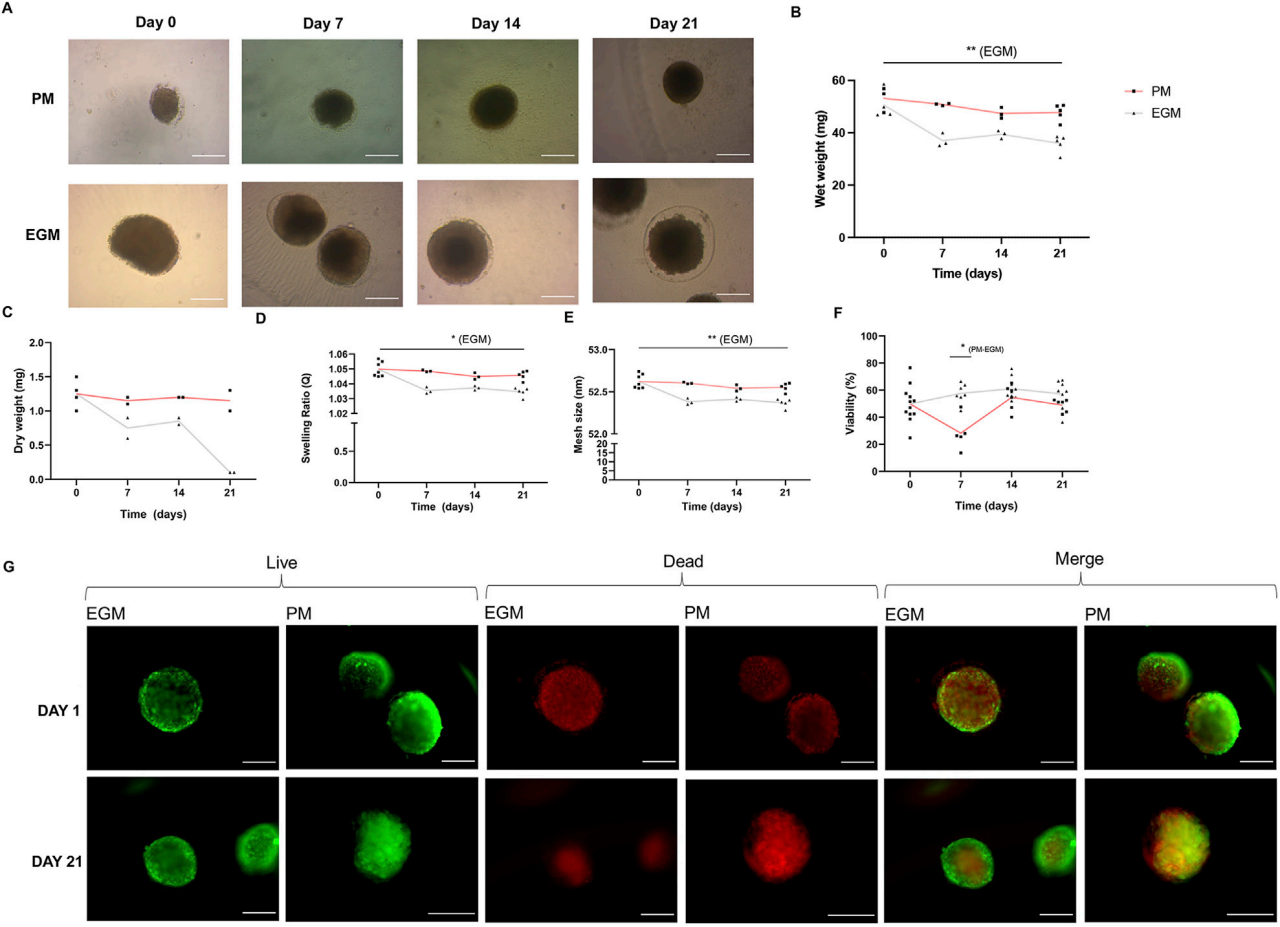
CaCl_2_-polymerized hydrogel entrapped spheroids. **(A)** Bright-field microscopic aspect of encapsulated spheroids. **(B)** Wet weight. **(C)** Dry weight. **(D)** Swelling Ratio. **(E)** Mesh size. **(F)** Spheroid viability, assessed through Live/Dead assay, over 21 days of experiment. **(G)** Representative Live/dead assay images. *p < 0.05, ** p < 0.01. Scale bar = 200 µm.

Following encapsulation, MDSC spheroids were cultured within the same alginate hydrogel formulation for 21 days in both PM and EGM (Figure 4A). Initial assessment of wet weight before immersion in specific culture media revealed a mean of 51.714 ± 4.972 mg for both PM and EGM on day 0, already 71%–106% heavier than clean hydrogels. By the end of the 21 days, the mean dry weight for PM was 47.80 ± 3.077 mg, and for EGM, it was 35.92 ± 3.226 mg (Figure 4B). Serial assessments of wet and dry weights demonstrated stability in PM and instability in EGM, with a decreasing trend (Figures 4B,C). This behavior became more pronounced over time (Figures 4B,C, p < 0.05 for EGM day 0 versus 21). Consequently, the swelling ratio of the alginate spheres cultivated in EGM also decreased over time (Figure 4D, p < 0.05 for EGM day 0 versus 21).

Finally, the mesh size of spheroids cultured in EGM significantly decreased by day 21 compared to its initial size, reaching a mean of 52.355 ± 0.051 nm, indicating a reduction of 0.4% (Figure 4E, p < 0.05 for EGM day 0 versus 21). In contrast, the PM group’s mesh size experienced a minimal reduction of 0.13% from its initial mean of 52.623 ± 0.084 nm.

The Live/Dead assay of spheroids within the hydrogel demonstrated initial viabilities of 49.778% ± 13.347% for both groups, assessed before immersion in specific culture media (Figures 4F,G). The viability of PM and EGM groups on day 21 revealed means of 48.905% ± 4.622% and 57.11% ± 10.682%, respectively. Although the 14.72% increase in viability for the EGM group was not statistically significant (Figures 4F,G), EGM viability was significantly superior to PM on day 7 (Figure 4E, p < 0.05 for day 7 p.m. versus EGM). This viability assay thus revealed a stability and growth pattern of EGM-cultured spheroids over time, contrasting with PM-cultured spheroids.

### 3.5 Encapsulated MDSC-VEGF spheroids are capable of retaining cell viability and are valid for *in vivo* assays

Based on the findings that an endothelial microenvironment (EGM) enhances MDSC stemness (Supplementary Figure S1C) and that direct co-culture with endothelial cells (HUVECs) improves spheroid viability (Figures 2E,F), we hypothesized that inducing an endothelial-like phenotype in the MDSCs themselves could be a superior and more translatable strategy.

To test this, we pre-treated MDSCs with VEGF (50 ng/mL) to promote endothelization prior to spheroid formation as previously described (Verma et al., 2024). We then generated therapeutic spheroids using a 95:5 mixture of naïve MDSCs and VEGF-pre-treated MDSCs (hereafter referred to as MDSC-VEGF), mirroring the optimal ratio used in the MDSC/HUVEC co-cultures.

**FIGURE 5:**
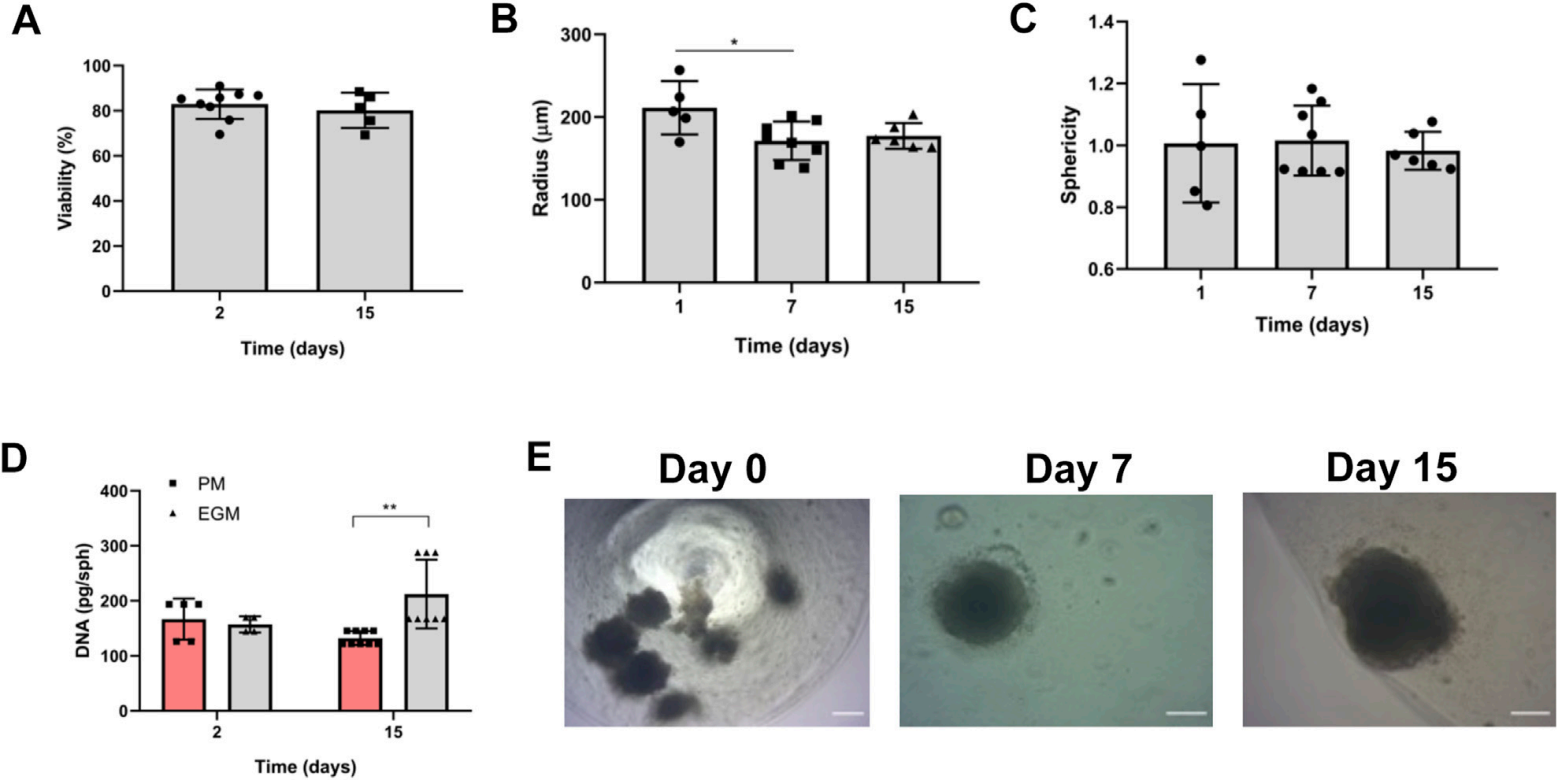
Characterization of MDSD/MDSC-VEGF spheroids. Alginate hydrogels containing 10 Spheroids each (10^5^ cells) were cultivated for 15 days. **(A)** Viability was evaluated by Live/Dead assay. **(B)** Radius. **(C)** Sphericity. **(D)** Proliferation was performed with Quant-iT™ PicoGreen™ assay. **(E)** Bright-field microscopic aspect of encapsulated spheroids. *p < 0.05, **p < 0.01. Scale bar = 200 µm.

MDSC-VEGF spheroids were encapsulated as the therapeutic vehicle in the characterized alginate hydrogel, with 10 spheroids per gel, cultivated in EGM for 15 days, and serially assessed for viability, through two different methods. Live/Dead viability revealed that all spheroids were able to preserve viability (Figure 5A, day 2%–82.95% ± 6.551%, day 15%–80.21% ± 7.831%), and exhibited the standard morphological measurements behavior (Figures 5B,C, radius and sphericity) as previously characterized (Figures 2B,C). Quantification of DNA per spheroid revealed an increase in genetic material per spheroid in EGM-cultured spheroids (Figure 5D, day 2–157.057 ± 14.632 pg, day 15–212.273 ± 62.466 pg, p < 0.05, for EGM day 2 versus day 15). Bright-field microscopic aspect of hydrogels is shown (Figure 5E).

### 3.6 Delivery of therapeutic alginate encapsulated spheroids reduces fibrosis and inflammation post-VML

**FIGURE 6:**
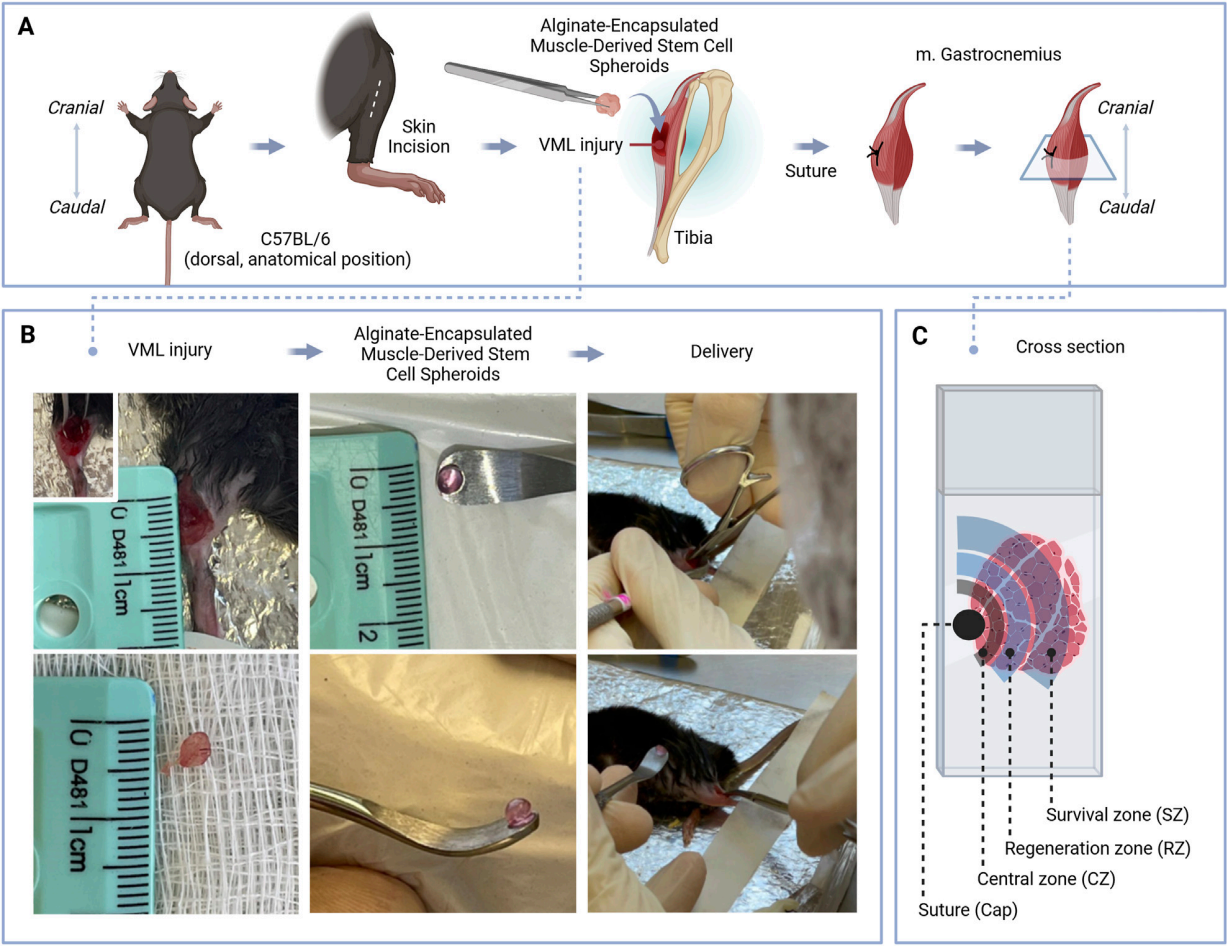
Overview of the experimental design and histological sampling strategy. **(A)** Schematic of the volumetric muscle loss (VML) model and treatment approach. Anesthetized C57BL/6 mice underwent a VML injury created with a 4 mm biopsy punch on the left gastrocnemius muscle. Alginate-encapsulated muscle-derived stem cell spheroids (10 per construct) were prepared using low molecular weight (LMW), non-oxidized alginate and maintained in EGM culture medium until implantation. **(B)** Intraoperative images illustrate the VML lesion, preparation of spheroid-laden alginate constructs, and delivery to the injury site using gentle Kelly retraction and curved dissector guidance for accurate placement. **(C)** Histological cross-sections were obtained from the central region of the injury site, centered on the suture site. The injury was divided into defined zones for analysis: central zone (CZ), regeneration zone (RZ), and survival zone (SZ), excluding the epimysial cap and surrounding tissue.

**FIGURE 7:**
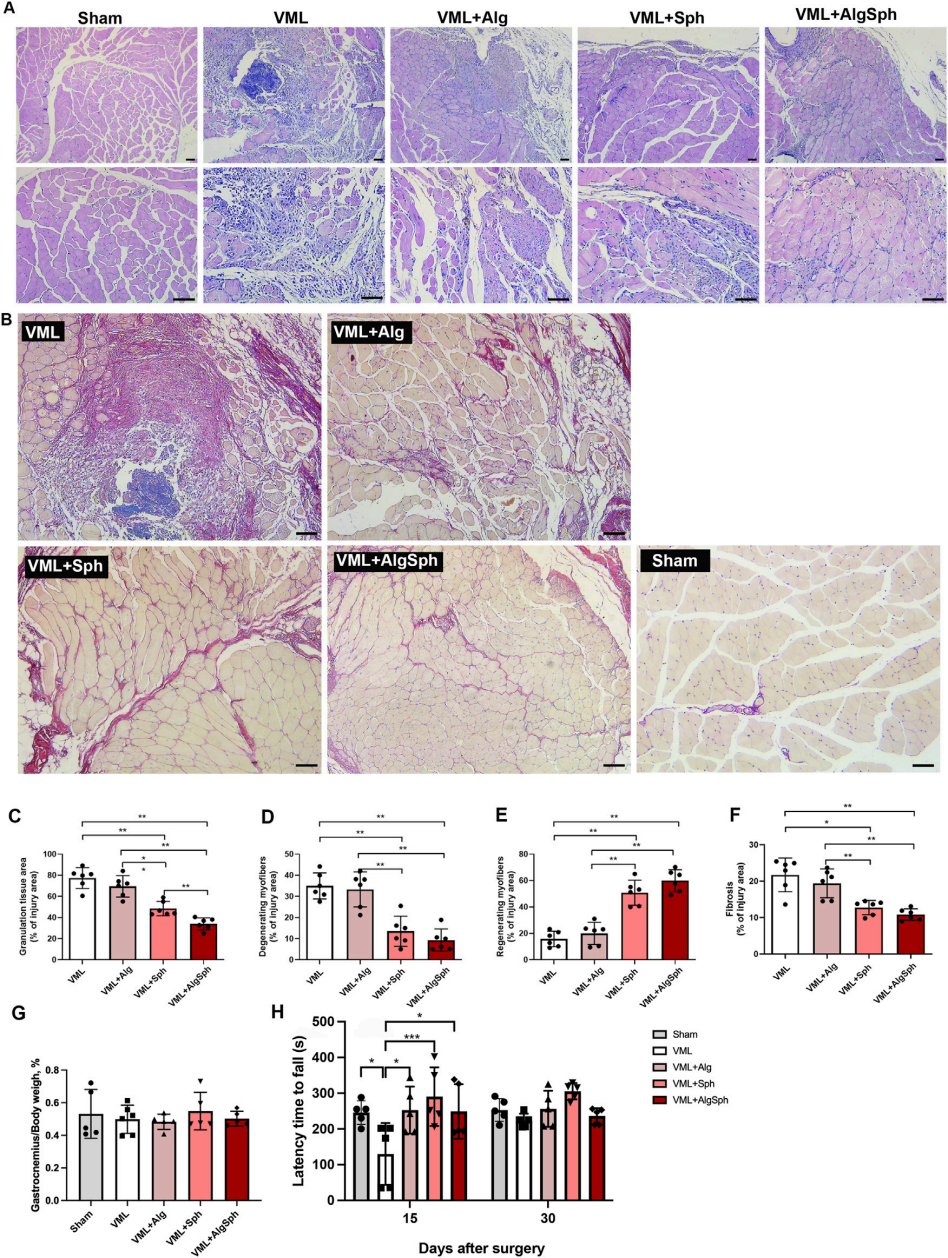
Histomorphometry and functional analysis of therapeutic strategies to VML. **(A)** Representative 100x and 200x amplification images of injury site Hematoxylin/Eosin staining histology, evidencing inflammatory infiltration, myocyte degeneration and granulation tissue. **(B)** Quantitative analysis of granulation tissue. **(C)** Degenerating. **(D)** Regenerating myofibers, rationalized per region of interest of injury area. **(E)** Representative 100x amplification images of injury site Picrosirius Red staining histology. **(F)** Total quantitative analysis of fibrotic area, per surgery group. **(G)** Ratio of mice gastrocnemius to body weight per surgery group. Quantification normalized to each sample’s injury area (ROI defined in 2.9). Sham is displayed as representative images only because a ‘% of injury area’ is not meaningful in the absence of a lesion; normalization for Sham is therefore not computed **(H)** Rota-Rod performance. Sham, VML = Injury non treated, VML+Alg = VML + Clean Alginate Hydrogel Delivery, VML+Sph = VML + Delivery of 10 unencapsulated spheroids, VML+AlgSph = VML + Delivery of alginate hydrogel containing 10 encapsulated spheroids. *p < 0.05. Scale bar = 50 µm. Group sizes were n = 6, except SHAM (n = 5) due to one pre-surgical death.

To evaluate the therapeutic efficacy of alginate-encapsulated spheroids in a muscle injury model of VML, we performed a surgical protocol involving partial removal of the gastrocnemius muscle (Figure 6). Subsequently, mice were categorized into different groups based on their therapeutic treatment. These groups included mice receiving therapeutic spheroids, which were characterized in the previous section, included spheroids alone (VML+Sph), hydrogel alone (VML+Alg), spheroids encapsulated by alginate (VML+AlgSph) and VML. Histomorphometric investigations were conducted on the injured gastrocnemius muscle, focusing on regions of interest (ROI) under bright-field microscopy following Hematoxylin and Eosin (H&E) staining (Figures 6C, 7; Supplementary Figure S2).

Mice receiving therapeutic spheroids (VML+Sph and VML+AlgSph) demonstrated the lowest quantifications of granulation tissue per injury area, with therapeutic encapsulated spheroids (VML+AlgSph) showing significantly less granulation tissue than spheroids alone (Figures 7A–C; 48.53% ± 6.79% versus 34.08% ± 5.637%, VML+Sph versus VML+AlgSph, respectively, p < 0.05). Additionally, mice receiving therapeutic encapsulated spheroids presented with the fewest number of degenerating myofibers per injury area, compared to the VML group and VML+Alg groups (Figures 7A–D; 34.97% ± 6.173% for VML, 33.32% ± 8.270% for VML+Alg, 9.317% ± 5.277% for VML+AlgSph, p < 0.05).

Conversely, mice receiving spheroids exhibited the highest quantities of regenerating myofibers per injury area (Figure 7E; 15.78% ± 5.885% for VML, 1997% ± 8.376% for VML+Alg, 50.85% ± 9.452% for VML+Sph, 5988% ± 8.332% for VML+AlgSph; p < 0.05). Bright-field microscopy of fibrotic regions of interest following Picrosirius red (PSR) staining is displayed in Figures 7B–F. Quantification of fibrotic area per ROI revealed that mice receiving spheroids (VML+Sph and VML+AlgSph) had the lowest percentages of fibrosis, though they were not statistically different from each other (Figures 7B–F; 21.73% ± 4.622% for VML, 19.39% ± 3.959% for VML+Alg, 12.77% ± 1.970% for VML+Sph, 10.84% ± 1.566% for VML+AlgSph, p < 0.05).

No significant differences in the gastrocnemius muscle to mice body weight ratio were detected (Figure 7G). Additionally, the Rota-Rod test showed that mice receiving spheroids alone or encapsulated by alginate performed significantly better than the VML group 15 days after the injury (Figure 7H, p < 0.05). Curiously, the group injected only with alginate also showed an improved performance at the Rota-Rod, compared to VML group (Figure 7H, p < 0.05). After 30 days there was no difference among the groups.

## 4 Discussion

VML remains a rare but significant cause of skeletal muscle tissue loss and atrophy (Garg et al., 2015). Current clinical treatments are limited in their ability to regenerate damaged muscle and fully restore tissue function, highlighting the need for novel muscle regeneration strategies (Eugenis et al., 2021; Haller et al., 2025). Cell therapy using MDSC offers a promising approach for VML treatment; however, poor cell viability following transplantation continues to represent a major challenge for the success of cell-based regenerative therapies (Eugenis et al., 2021). The present study evaluated the therapeutic potential of MDSC spheroids encapsulated within alginate hydrogels for promoting muscle regeneration and functional recovery in a murine VML model. To our knowledge, this is the first study to explore the use of alginate-encapsulated MDSC spheroids specifically for skeletal muscle regeneration following VML injury.

MDSC possesses a strong proliferative capacity, sustained self-renewal, and multipotent differentiation potential, setting them apart from other muscle progenitors such as satellite cells (Relaix et al., 2021). Importantly, MDSCs exhibit remarkable resilience in hostile microenvironments, including enhanced survival under hypoxic conditions and resistance to oxidative stress—features that contribute to their superior engraftment efficiency *in vivo* compared to other muscle stem cell populations (Usas and Huard, 2007; Vella et al., 2011; Matthias et al., 2018). In addition to their myogenic capacity, MDSCs actively contribute to the regenerative environment by promoting both neovascularization and neurodegeneration at the injury site, processes that are essential for functional tissue integration and recovery (Ota et al., 2011; Lavasani et al., 2014). These combined characteristics highlight the therapeutic potential of MDSCs as a robust cell source for addressing volumetric muscle loss and other severe muscle-related injuries.

Stem cell survival and sustained secretion of growth factors are crucial for successful tissue regeneration. In this context, we demonstrated that MDSCs cultured in EGM exhibit significantly enhanced expression of *Sca-1*, a well-established marker of stemness, compared to traditional proliferation media (Epting et al., 2008). Previous studies suggest that endothelial signaling plays a critical role in stem cell regulation, with VEGFA being crucial for maintaining muscle stem cell (MuSC) quiescence (Verma et al., 2018; 2024). Notably, the VEGFA-FLT1 pathway has been shown to promote MuSC survival by suppressing apoptosis (Verma et al., 2024). Additionally, myogenic progenitors derived from pluripotent stem cells exhibit enhanced contractility when differentiated in EGM-2-supplemented conditions (Xu et al., 2019).

In this study, we used a spheroid-based cellular delivery method, utilizing alginate hydrogel as a vehicle. Two key reasons for choosing this system include the possibility of culturing a large amount of stem cells in a 3D environment through spheroids, and the capability of its development, with preserved viability, in a tunable biomaterial with low levels of rejection (Matthias et al., 2018).

The use of 3D spheroid culture represents a significant advancement over traditional 2D cell culture methods. Spheroids enhance cell-cell interactions and mimic the native tissue architecture, promoting stem cell maintenance and differentiation (Stuart et al., 2017; Gahlawat et al., 2024).

Interestingly, we observed that coculture spheroids containing both MDSCs and HUVECs exhibited increased viability compared to MDSC-only spheroids, especially at early time points. These results suggest beneficial effects of endothelial cells within spheroids, potentially through paracrine signaling that enhances stem cell viability and function. This coculture strategy has recently been shown to support vascularization, survival, and improved functionality of engineered tissues (Schwaerzer, 2023).

In terms of morphology and growth dynamics, MDSC spheroids displayed a bimodal growth pattern characterized initially by size reduction followed by recovery, while coculture spheroids exhibited intermediate and eventually similar behavior to MDSC spheroids. These data further illustrate the adaptability and robustness of spheroid-based culture methods, supporting their suitability for therapeutic applications. Despite these variations, all spheroids-maintained viability above 80%, with MDSC/HUVEC spheroids showing higher viability compared to MDSC alone at critical time points.

Encapsulation of MDSC spheroids in alginate hydrogels provided a supportive microenvironment that preserved cell viability, promoted genetic material retention, and facilitated sustained therapeutic activity. Encapsulated MDSC spheroids cultivated in EGM resulted in progressive reductions in wet weight, swelling ratio, and mesh size—likely reflecting interactions between alginate polymers and the cells. Notably, encapsulated MDSC spheroids cultivated in EGM maintained viability around 60% over a 21-day culture period. Conversely, spheroids cultivated in PM presented a lower viability over time (Figures 4F,G). These findings are consistent with previous reports in the literature, although those studies utilized mesenchymal stem cells rather than MDSCs (Gionet-Gonzales et al., 2021).

To enhance cell viability and regenerative potential, we engineered composite spheroids containing 95% MDSCs maintained in EGM and 5% MDSCs preconditioned with VEGF (50 ng/mL) for 7 days before spheroid assembly. The 5% fraction was guided by Deegan et al. (2019), who showed that adding 5% HUVECs to mesenchymal spheroids improves viability and paracrine output; here, VEGF-preconditioned MDSCs were used to emulate this trophic/endothelial support. This strategy is further supported by evidence that VEGF signaling is critical for MuSC survival and regenerative competence—specifically, the VEGFA–FLT1–AKT1 axis in ischemic niches (Verma et al., 2024). Consistent with this rationale, composite spheroids exhibited higher 15-day viability accompanied by increased DNA content, indicating sustained proliferation/metabolic activity. Collectively, these data suggest that alginate-based encapsulation, particularly when paired with VEGF preconditioning, effectively supports long-term cellular function—an essential prerequisite for regenerative applications.

In addition to enhancing cell viability, alginate hydrogels offer key physicochemical advantages, including mechanical stability and bioactive factor retention, both of which are critical for maintaining therapeutic efficacy *in vivo*. When cultured in PM or EGM, alginate hydrogels preserved their physical properties, with rheological characteristics closely resembling those of native human skeletal muscle tissue (Green et al., 2012).

The loss of a well-organized extracellular matrix (ECM) in VML represents a major obstacle to cell retention and functional tissue regeneration (Langridge et al., 2021). In this setting, tissue-engineered scaffolds such as hydrogels play a pivotal role by mimicking native ECM properties, supporting cell adhesion, and enabling the controlled release of growth factors (Lev and Seliktar, 2018). Hydrogel-based scaffolds, in particular, are advantageous due to their capacity to absorb biological fluids (El-Sherbiny and Yacoub, 2013), adapt to the geometry of the defect site, and promote localized delivery of therapeutic agents. These features make alginate hydrogels a versatile platform for improving cell-based regenerative strategies, especially in complex environments like VML.

In our study, the therapeutic efficacy of encapsulated MDSC spheroids was validated in a murine model of VML. Mice treated with encapsulated spheroids exhibited significant reductions in granulation tissue, degenerating myofibers, and fibrosis compared to control groups. These results are consistent with prior studies demonstrating that the sustained release of anti-inflammatory and pro-regenerative factors from encapsulated stem cells can effectively modulate the local microenvironment, reducing fibrosis and inflammation (Gionet-Gonzales et al., 2023; Wu et al., 2025). These findings are also consistent with a previous study from our group, which demonstrated reduced fibrosis and fewer degenerating fibers in a muscle laceration model treated with alginate injection (Felipone et al., 2025). This combination of decreased fibrotic tissue formation, increased myofiber regeneration, showed the sustained viability and activity of the implanted MDSC spheroids. Together, these findings highlight the potential of alginate-encapsulated spheroid therapy as a promising strategy for restoring structure and function in severe muscle injuries such as VML.

Although immunohistochemical markers for macrophages or mast cells were not included, the quantitative analysis of granulation tissue and fibrosis provided robust insight into the inflammatory and regenerative status of the injured muscle. Our histomorphometric data, demonstrating progressive reductions in both parameters, particularly in the VML+Sph and VML+AlgSph groups—indicate a transition from persistent inflammation toward effective tissue remodeling. These findings align with previous studies showing that modulation of chronic inflammatory pathways facilitates regeneration while limiting scar formation. For example, Mourkioti and Rosenthal (2005) demonstrated that IGF-1 overexpression in dystrophic muscle reduces fibrosis and enhances repair through TGF-β signaling modulation. Similarly, Martins et al. (2020), Martins et al. (2023) reported that targeting macrophage-driven inflammation or TGF-β1 activity results in decreased granulation tissue and fibrosis, supporting functional muscle recovery. In line with this, Rodriguez Ayala et al. (2025) showed that biomaterial-based immunotherapies in VML models attenuate chronic inflammation and fibrosis while promoting a regenerative microenvironment conducive to myofiber restoration. Altogether, these data support the view that reducing granulation tissue and fibrotic burden reflects a favorable shift in the muscle healing trajectory. The improved regeneration outcomes observed in the encapsulated spheroid group can be attributed to the combined effects of stem cell viability, growth-factors secretion, and the supportive hydrogel environment. These factors likely acted synergistically to create a pro-regenerative microenvironment within the injured muscle tissue.

Regarding the functional outcomes, animals treated with encapsulated MDSC spheroids demonstrated superior recovery, as evidenced by significantly improved performance in the Rota-Rod test. Interestingly, mice treated with either spheroids alone or alginate alone also showed better performance compared to the untreated VML group. This highlights a limitation of our study, as functional recovery was assessed exclusively using the Rota-Rod test. Although this test is valuable for evaluating gross motor coordination and endurance, it does not comprehensively assess muscle strength, contractile capacity, or fine motor skills. Future studies should incorporate additional functional assays—such as grip strength measurements, gait analysis, or electrophysiological testing—to enable a more complete evaluation of muscle performance.

Recent studies have highlighted complementary dimensions of VML recovery that extend beyond structural regeneration. For example, Bruzina et al. (2024) reported that VML impairs skeletal muscle metabolic flexibility and that this effect is only moderately exacerbated by physical activity restriction. Although we did not investigate metabolic outcomes, our finding of reduced fibrosis and enhanced regeneration may indirectly support conditions favorable to preserving muscle function and metabolism. Similarly, Greising et al. (2018) showed that early rehabilitative strategies, such as passive range of motion and electrical stimulation, can enhance muscle strength and modulate tissue biomechanics, even without significantly altering collagen content. While such approaches were not applied in the present study, their findings point to the potential benefit of combining regenerative biomaterials, such as our VEGF-preconditioned MDSC spheroids, with physical rehabilitation to synergize structural and functional recovery. Future studies integrating metabolic and biomechanical analyses would be valuable for understanding the full regenerative impact of such therapies.

Another relevant aspect concerns the use of calcium alginate, a known hemostatic material (Xie et al., 2022; Zheng et al., 2022). Calcium alginate can facilitate the entry of calcium ions into the wound via ion exchange with sodium ions present in the blood. This, in turn, stimulates the activation of clotting factors VII, IX, and X, promotes platelet aggregation, initiates the coagulation cascade, and accelerates hemostasis (Wu et al., 2020). Moreover, calcium alginate exhibits favorable properties such as high-water absorption capacity, oxygen permeability, mucosal adhesion, and excellent biocompatibility (Xie et al., 2022; Zheng et al., 2022). These characteristics may contribute to the improved functional outcomes observed in the group treated solely with alginate, compared to the untreated VML group.

Our findings provide proof-of-concept that MDSC spheroids encapsulated in alginate hydrogels can support structural regeneration and functional improvement in a murine VML model. The use of endothelial media to enhance stemness and the encapsulation strategy to preserve viability and paracrine activity establish a biologically plausible and translationally relevant platform. A limitation of our work is that we did not include a control group composed of 100% unconditioned MDSC spheroids embedded in alginate hydrogel. Although *in vitro* viability assays suggested that fully unconditioned MDSC spheroids would perform poorly, this assumption cannot substitute for direct *in vivo* evaluation. As elegantly demonstrated by Deasy et al. (2009), *in vitro* proliferation or survival of VEGF-modified myogenic cells does not necessarily predict their regenerative performance *in vivo*. Consequently, within the current experimental design we cannot determine whether VEGF preconditioning truly confers a superior therapeutic effect compared with unconditioned MDSCs. Future studies will need to directly compare VEGF-preconditioned and fully unconditioned MDSC spheroids, incorporate additional functional and mechanistic readouts (e.g., angiogenic and myogenic gene expression profiles, immune modulation, and metabolic parameters), and evaluate scalability and long-term biocompatibility in order to determine whether VEGF preconditioning confers a true therapeutic advantage and to advance this approach toward clinical application.

## 5 Conclusion

This work provides proof-of-concept that alginate-encapsulated MDSC spheroids, including a small VEGF-preconditioned subfraction, can modulate histological indices of repair and support early functional recovery after VML. We do not intend to assert *in vivo* superiority of VEGF preconditioning over unconditioned MDSCs; rather, we propose this combined cell–biomaterial–pharmacology approach as a promising platform that should be further examined in future studies specifically designed and powered to address that question.

## Data Availability

The author selected the following statement: The datasets presented in this study can be found in online repositories. The names of the repository/repositories and accession number(s) can be found below: Harvard Dataverse, https://doi.org/10.7910/DVN/YUNDWV.
