# Supplementary material for: Alginate-encapsulated muscle-derived stem cell spheroids promote muscle regeneration in a murine model of volumetric muscle loss
Source: Front Pharmacol. 2025 Dec 12;16:1657563. doi: 10.3389/fphar.2025.1657563 (PMC12740859; doi:10.3389/fphar.2025.1657563)
Supplement: Supplementary file 1 [file DataSheet1.pdf]

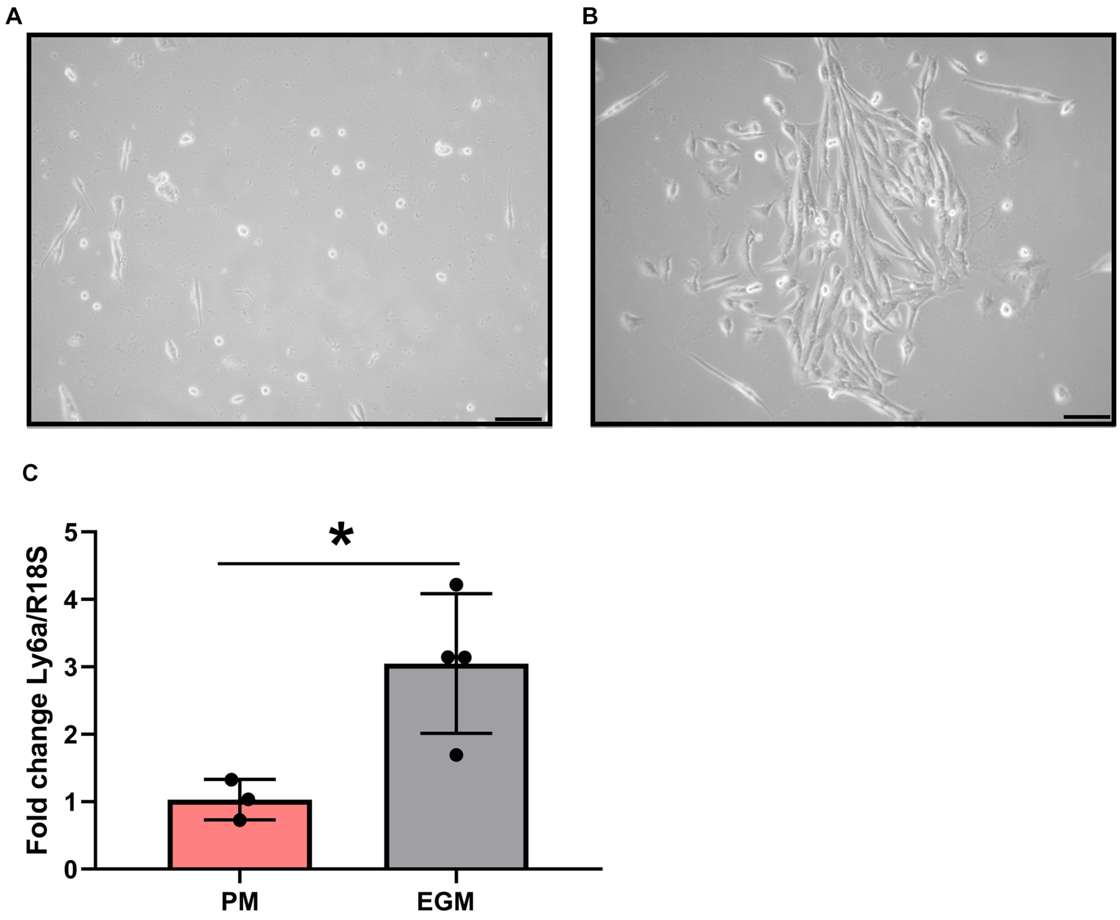

Supplementary figure 1: Characterization of MDSC population. A. MDSC extracted from C57Bl/6 mice were cultivated in proliferation media (PM) on low confluency. B. MDSC were allowed to achieve high confluency before the real-time PCR (qPCR) assay. C. MDSC were cultured in PM (control) and endothelial growth media (EGM) until passages 4-6 (high confluency), and genetic material was extracted for qPCR analysis, assessing the expression of *Ly6a* (Sca-1). Scale bar = 100 μm. n=3-4.

Supplementary figure 2: Representative cross-section of the gastrocnemius muscle 30 days post-injury. The ablation and suture area is outlined with a dashed red line. Distinct histopathological zones are identified: Survivor Zone (SZ), Regeneration Zone (RZ), Central Zone (CZ), and Fibrillar Cap from the suture (Cap) (*top*). Key histological features are highlighted (*bottom*): regenerating fibers with central nuclei (arrow), degenerating fibers (arrowhead), and granulation tissue (asterisk).

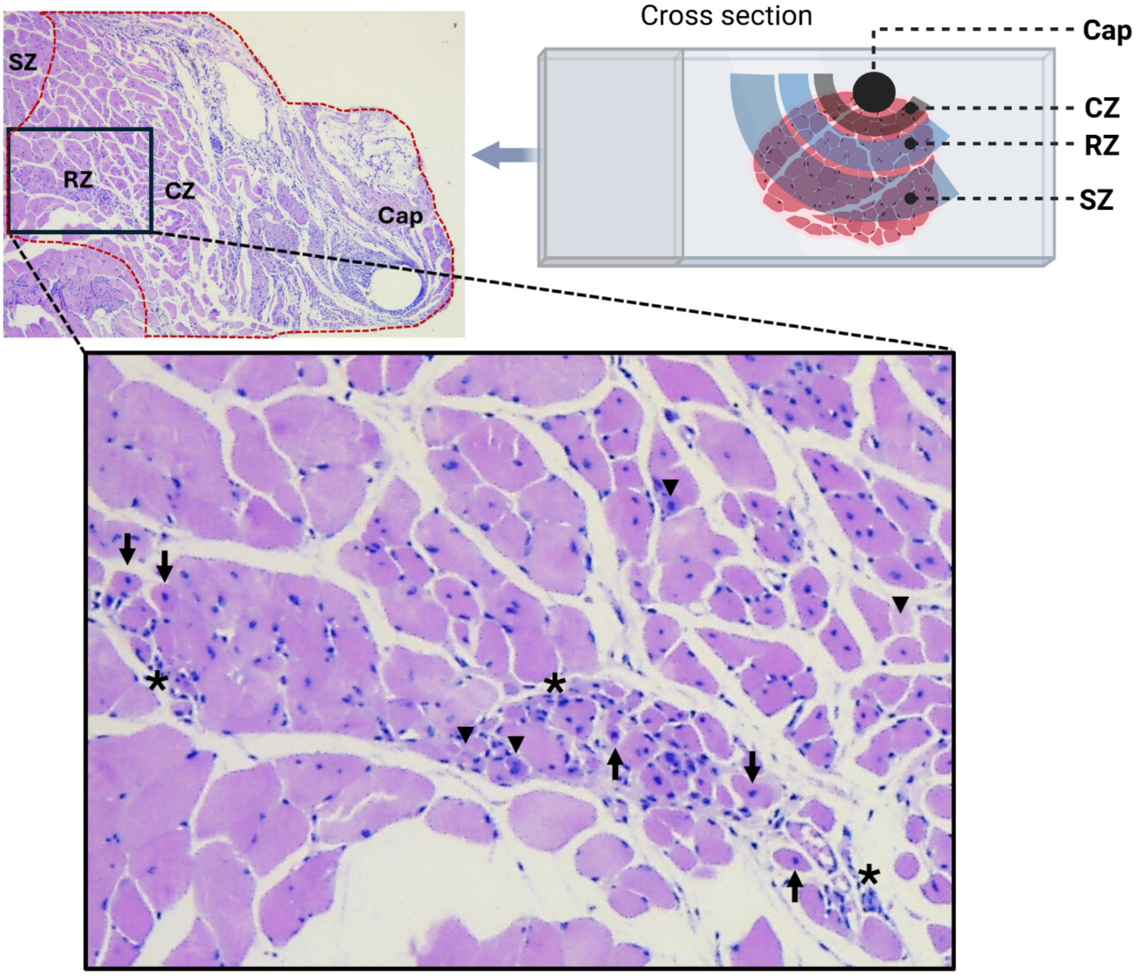

Supplementary table 1. Criteria adopted for histomorphology analysis of HE-stained skeletal muscle tissue.

Classification	Histomorphology findings	Reference
**Degeneration**	Cellular swelling, hypereosinophilia, vacuolization, fibre fragmentation and rupture.	[1;2; 3]
**Regeneration**	Fibres with centralized nucleus myocytes; Basophilic staining (active regeneration).	[1;2; 3]
**Granulation tissue**	Scattered abundance of eosinophilic fibroblasts (cytoplasmic fibrillar system) surrounded by mononuclear inflammatory cells.	[4; 5; 6]
**Muscle atrophy**	Reduction of myofiber diameter and hypereosinophilic sarcoplasm.	[1;2;3]
**Inflammation**	Presence of Polymorphonuclear neutrophilic (PMN), leukocyte and/or infiltrated leucocytes with varying morphological patterns.	[2;3;7]
**Fibrosis**	Increased number of reactive fibroblasts with prominent vesiculated nuclei; Increased amount of pale eosinophilic fibrillary material (collagen deposition) separating and / or surrounds adjacent myofibers.	[3;8]

Reference

1. Sayers SP, Hubal MJ. Histological, Chemical and functional manifestations of muscle damage. Tiidus PM, editor. (ed.) Skeletal muscle damage and repair. Human Kinetics, Champaign; 2007.

2. Swery CA, Goebel HH. General pathology of muscle disease. In: Goebel HH, Swery CA, Weller RO, editors. Muscle disease. Pathology and genetics. 2nd ed. Oxford: Wiley Blackwell; 2013. p. 19–38. Doi: 10.1002/9781118635469.ch3.

3. Michael Kyba (ed.), Skeletal Muscle Regeneration in the Mouse: Methods and Protocols, Methods in Molecular Biology, vol. 1460, DOI 10.1007/978-1-4939-3810-0_5, Springer Science Media New York 2016

4. Gabbiani G, Hirschel BJ, Ryan GB, Statkov PR, Majno G. Granulation tissue as a contractile organ. A study of structure and function. J Exp Med. 1972 Apr 1;135(4):719-34. doi: 10.1084/jem.135.4.719.

5. Mann, C.J., Perdiguero, E., Kharraz, Y. *et al.* Aberrant repair and fibrosis development in skeletal muscle. *Skeletal Muscle* **1**, 21 (2011). https://doi.org/10.1186/2044-5040-1-21

6. Lehto, M., Sims, T.J. & Bailey, A.J. Skeletal muscle injury—molecular changes in the collagen during healing. *Res. Exp. Med.* **185**, 95–106 (1985). https://doi.org/10.1007/BF01854894

7. Tidball JG. Inflammatory processes in muscle injury and repair. Am J Physiol Regul Integr Comp Physiol. 2005 Feb;288(2):R345-53. doi: 10.1152/ajpregu.00454.2004.

8. Mahdy MAA. Skeletal muscle fibrosis: an overview. Cell Tissue Res. 2019 Mar;375(3):575-588. doi: 10.1007/s00441-018-2955-2.
